# Management of acute uncomplicated diverticulitis and adherence to current guidelines—a multicentre SNAPSHOT study

**DOI:** 10.1007/s00384-024-04701-z

**Published:** 2024-08-08

**Authors:** Helene R. Dalby, Alessandro Orrú, Frida Sundh, Pamela Buchwald, Fredrik Brännström, Bengt Hansske, Staffan Haapaniemi, Maziar Nikberg, Abbas Chabok

**Affiliations:** 1 Colorectal Unit, Department of Surgery, and Centre for Clinical Research, Uppsala University, Västmanlands Hospital Västerås, Västerås, Sweden; 2 Department of Surgery, Randers Regional Hospital, Randers, Denmark; 3 Division of Surgery, Danderyd University Hospital, Stockholm, Sweden; 4 Department of Surgery, Skåne University Hospital, Lund University, Malmö, Lund Sweden; 5 Department of Surgery, Capio S:T Görans Hospital, Stockholm, Sweden; 6 Department of Surgery, Torsby Hospital, Torsby, Region Värmland Sweden; 7 Department of Surgery, Norrköping, Sweden; 8 Center for Clinical Research, Uppsala University, Västerås, Region Västmanland Sweden

**Keywords:** Acute uncomplicated diverticulitis, Antibiotic treatment, Clinical guidelines, Clinical trials

## Abstract

**Purpose:**

To explore whether previous participation in clinical studies increases adherence to management guidelines in acute uncomplicated diverticulitis (AUD).

**Methods:**

This retrospective cohort study was designed to give a SNAPSHOT of the management of AUD at six hospitals, three of which had participated in the AVOD trial comparing antibiotic versus non-antibiotic treatment of AUD. Patients with AUD were included from March 2019 through June 2020 and followed for 90 days. The primary outcome was treatment of AUD categorised by antibiotic treatment and inpatient or outpatient management compared between AVOD and non-AVOD hospitals. Descriptive statistics were compiled, and differences between hospitals were assessed with Pearson’s chi-squared test.

**Results:**

The cohort included 449 patients with AUD of which 63% were women and the median age was 63 (IQR: 52–73) years. Patient characteristics were comparable across the hospitals. Antibiotics were administered to 84 (19%) patients and 113 (25%) patients were managed as inpatients. Management varied significantly between AVOD and non-AVOD hospitals. The mean proportion of patients treated with antibiotics was 7% at AVOD hospitals compared to 38% at non-AVOD hospitals (*p* < 0.001). The mean proportion of in-hospital management was 18% at AVOD hospitals versus 38% at non-AVOD hospitals (*p* < 0.001).

**Conclusion:**

Most patients with AUD were managed according to current guidelines. However, the management varies between hospitals and previous participation in clinical studies may increase knowledge of and adherence to guidelines.

**Supplementary Information:**

The online version contains supplementary material available at 10.1007/s00384-024-04701-z.

## Introduction

Diverticulosis is one of the most common diseases of the gastrointestinal tract in Western countries, and incidence has increased by 32–50% during recent decades [1, 2]. Most diverticulosis cases remain asymptomatic, but around 5% progress to symptomatic disease [3]. The most common presentation of symptoms is acute uncomplicated diverticulitis (AUD), and only a few patients develop complications such as abscess or perforation [4]. Traditionally, treatment included hospitalisation, antibiotics, analgetics, and bowel rest [5, 6]. During the early 2010s, several landmark studies established the evidence for current guidelines for AUD [7, 8]. First, two randomised controlled clinical trials (RCTs) in Sweden (AVOD [9]) and the Netherlands (DIABLO [10]) established that AUD can safely be managed without antibiotics with no increased risk of complications or recurrence. This has been confirmed in long-term follow-up [11, 12]. Second, outpatient management of AUD has proven to be safe in both the Spanish RCT DIVER trial and several cohort studies [13–16], even without antibiotic treatment [17].

Structured management guidelines can elevate the standard of care in clinical practice. Implementing results from clinical trials into guidelines and subsequently implementing these guidelines into clinical practice face substantial challenges. Studies of management of AUD have shown lower than expected compliance with guidelines that recommend treatment without antibiotics, indicating substantial challenges in implementing new guidelines [18, 19]. Presumably, management according to guidelines may be more common in hospitals that previously participated in clinical trials that formed the basis for current guidelines.

This study aimed to evaluate differences in the management of AUD across Swedish hospitals. We hypothesised that the management of AUD varies between hospitals and that previous participation in the AVOD trial would increase adherence to current guidelines.

## Materials and methods

### Design

This cohort study was designed to give a SNAPSHOT of current management practices of AUD at six emergency hospitals across Sweden. Participating hospitals included Malmö, Södertälje, Torsby, Danderyd, Norrköping, and Västerås of which the latter three participated in the AVOD study from 2003 to 2010 [9, 12]. The inclusion period was from March 2019 through June 2020. Patients were retrospectively identified based on ICD-10 codes (K573 and K579) at participating hospitals. Exclusion criteria included age below 18 years, patients hospitalised for complicated diverticulitis, diverticular bleeding, diverticular stenosis, or elective hospitalisation. The date of emergency hospital visit with AUD was the index date (index AUD). After identifying eligible patients, medical files were reviewed for study variables including baseline characteristics, follow-up measures, and outcomes. Patient follow-up extended for 90 days post-index AUD.

The study was approved by the Swedish Ethics Authority (No.: 2019–0383). The study was reported following the Strengthening the Reporting of Observational Studies in Epidemiology (STROBE) reporting guideline (Supplementary table) [20].

### Patient characteristics

Assessed variables included patient characteristics, detailed information about comorbidities, risk factors for complications to diverticulitis including use of immunosuppressives (e.g. immunotherapy), and history of diverticulitis with or without previous hospitalisation. Information recorded at the index AUD visit included subjective symptoms, clinical examination findings including abdominal tenderness, temperature, saturation, pulse, and blood pressure, laboratory results for inflammation markers including C-reactive protein (CRP), and results from computer tomography (CT).

### Index AUD

The primary outcome was the treatment at the index AUD visit categorised as treatment with or without antibiotics. Treatment was further classified based on whether it occurred in an outpatient setting or during hospitalisation. Management was categorised as outpatient if the patient was discharged from the emergency department within 24 h. For all hospitalisations, the treatments and length of stay were documented. Outcomes were compared among the participating centres and evaluated concerning previous participation in the AVOD study.

### Follow-up

During the 90-day follow-up period, new emergency hospital visits, i.e. revisits, were recorded. For patients with revisits, the following information was registered: if they had a CT scan, received antibiotic treatment, were managed as outpatients or hospitalised, and if any complications occurred. Moreover, it was registered if patients were referred for colonic investigations at the index AUD. If a colonic investigation was performed within the 90-day follow-up, the results were recorded.

### Statistical analysis

Descriptive statistics were compiled. Quantitative data were presented as median with interquartile (IQR) range and categorical data as absolute numbers and percentages. The difference in treatment strategy (with or without antibiotics and outpatient or inpatient management) between participating centres depending on previous participation in the AVOD study was evaluated using Pearson’s chi-squared test. Additionally, the association between treatment strategy and revisit within 90 days was investigated using Pearson’s chi-squared test. In a subanalysis, the treatment strategies for patients with CT-verified AUD were compared between AVOD and non-AVOD hospitals, excluding those patients who did not undergo at CT. The significance level was set at 0.05. Statistical analyses were performed using RStudio, version 2024.04.0 (Posit PBC).

## Results

### Patient characteristics

A total of 449 patients with AUD were included, of which 63% were women and the median age was 63 (IQR: 52–73) years. Of all patients, 151 (34%) had a history of diverticulitis. Patient characteristics, comorbidities, and potential risk factors were consistent across the hospitals. Selected patient characteristics are presented in Table 1.

**Table 1:** Characteristics of patients with acute uncomplicated diverticulitis at the time of index hospital visit

	AVOD overall	Danderyd	Norrköping	Västerås	Non-AVOD overall	Malmö	Södertälje	Torsby
	282 (63%)	123 (27%)	68 (15%)	91 (20%)	167 (37%)	86 (19%)	54 (12%)	27 (6%)
Sex								
Female	182 (65%)	81 (66%)	45 (66%)	56 (62%)	98 (59%)	58 (67%)	24 (45%)	16 (59%)
Male	100 (35%)	42 (34%)	23 (34%)	35 (38%)	68 (41%)	28 (33%)	29 (55%)	11 (41%)
Age*	62 (52–72)	62 (52–73)	66 (57–75)	59 (51–71)	63 (53–74)	64 (53–75)	59 (51–71)	71 (59–77)
BMI* *(unknown: 293)*	26 (23–29)	23 (21–27)	30 (30–30)	27 (24–29)	28 (25–32)	27 (24–32)	28 (25–31)	30 (28–39)
Smoking								
Active smoker	14 (5)	8 (7)	-	6 (6)	13 (8)	6 (7)	7 (13)	-
Never smoker	246 (87)	109 (89)	68 (100)	69 (76)	143 (86)	76 (88)	41 (76)	26 (96)
Previous smoker	22 (8)	6 (5)	-	16 (18)	11 (7)	4 (5)	6 (11)	1 (4)
Comorbidities								
Cardiovascular disease	38 (13)	14 (11)	10 (15)	14 (15)	46 (28)	32 (37)	11 (20)	3 (11)
Pulmonary disease	20 (7)	14 (11)	4 (6)	2 (2)	21 (13)	9 (10)	9 (17)	3 (11)
Diabetes mellitus	19 (7)	8 (7)	8 (12)	3 (3)	21 (13)	10 (12)	8 (15)	3 (11)
Risk factors								
Corticosteroids	10 (4)	5 (4)	4 (6)	1 (1)	4 (2)	-	-	4 (15)
Immunosuppressives	6 (2)	4 (3)	2 (3)	-	3 (2)	1 (1)	-	2 (7)
NSAIDs use	4 (1)	1 (1)	1 (2)	2 (2)	6 (4)	-	6 (11)	-
History of AD								
Yes	94 (33)	34 (28)	27 (40)	33 (36)	57 (34)	27 (32)	21 (39)	9 (33)
No	172 (61)	76 (62)	40 (59)	56 (62)	102 (61)	58 (67)	29 (54)	15 (56)
Unknown	16 (6)	13 (11)	1 (1)	2 (2)	8 (5)	1 (1)	4 (7)	3 (11)

*N* (%), *Median (IQR)

Findings at the index AUD visit are presented in Table 2. The median temperature was 37.1 (IQR: 36.7–37.6) °C and the median CRP was 66 (IQR: 34–105) mg/L, with no statistical difference between the hospitals. AUD was CT-verified in 369 (82%) patients. Among the 80 patients with no CT, 50 patients, 11% of all, had previous hospital contact with AUD. The proportion of patients with CT-verified AUD was 85% (*n* = 240) among AVOD hospitals and 77% (*n* = 129) among non-AVOD hospitals (*p* < 0.05).

**Table 2:** Findings at index hospital visit in patients with acute uncomplicated diverticulitis

	AVOD ﻿overall	Danderyd	Norrköping	Västerås	Non-AVOD ﻿overall	Malmö	Södertälje	Torsby
	282 (63%)	123 (27%)	68 (15%)	91 (20%)	167 (37%)	86 (19%)	54 (12%)	27 (6%)
Blood pressure								
Systolic* (mmHg)	143 (131–156)	141 (128–54)	147 (133–160)	144 (133–160)	140 (128–154)	136 (124–150)	142 (128–155)	147 (130–154)
Diastolic* (mmHg)	85 (76–93)	81 (75–89)	90 (79–98)	86 (77–93)	80 (71–90)	76 (68–87)	87 (78–100)	79 (69–84)
Pulse* (beats/min)	84 (74–94)	81 (73–88)	88 (78–97)	89 (75–97)	85 (77–98)	84 (76–94)	88 (79–100)	84 (79–95)
Temperature* (⁰C)	37.1 (36.7–37.5)	37.0 (36.6–37.3)	37.4 (36.9–37.5)	37.2 (36.7–37.6)	37.2 (36.7–37.6)	37.3 (36.7–37.7)	37.1 (36.7–37.7)	37.1 (36.5–37.3)
Inflammation markers								
CRP* (mg/L)	68 (35–102)	68 (37–101)	58 (28–80)	75 (41–113)	63 (31–107)	80 (37–124)	64 (42–97)	29 (16–58)
Leucocytes* (10^9^/L)	10 (8–12)	10 (8–13)	11 (9–12)	10 (8–12)	11 (9–13)	11 (9–13)	11 (9–13)	9 (8–12)
CT scan at index								
Yes	240 (85%)	106 (87%)	58 (85%)	76 (84%)	129 (77%)	62 (72%)	45 (83%)	22 (81%)
No	42 (15%)	17 (13%)	10 (15%)	15 (16%)	38 (23%)	24 (28%)	9 (17%)	5 (19%)

*N* (%), *Median (IQR)

### Index AUD

**Fig. 1:**
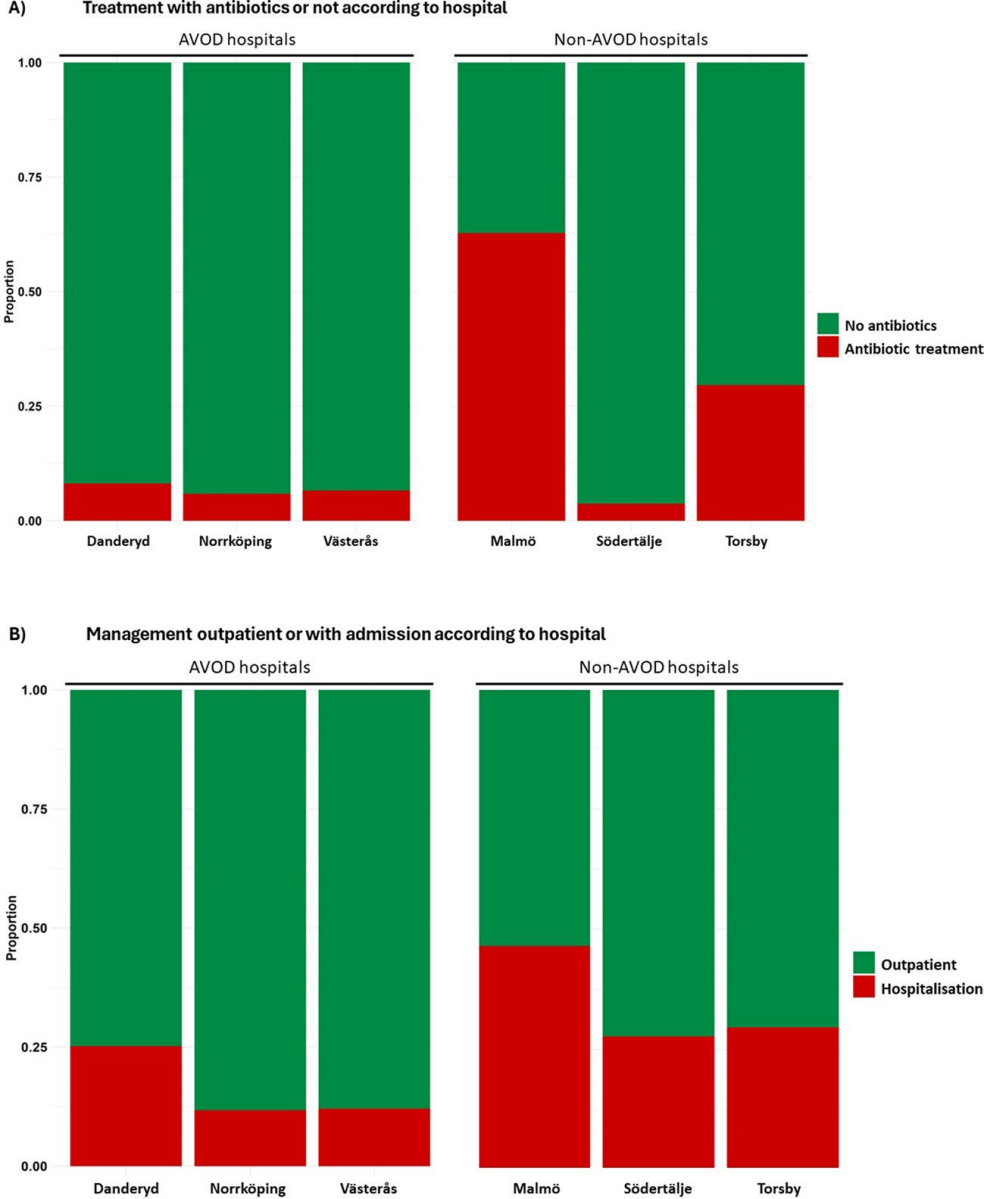
**A** Treatment with antibiotics or not according to hospital. **B** Management outpatient or with admission according to hospital

At the index AUD visit, 84 (19%) patients were treated with antibiotics. A total of 336 (75%) patients were managed as outpatients, while 113 (25%) were hospitalised (Table 3). The overall median length of stay for those hospitalised was 5 (IQR: 2–7) days. Among the patients managed as outpatients, 28 (8%) received antibiotic treatment, while 56 (50%) of the hospitalised patients received antibiotic treatment. Treatment regimens varied between AVOD and non-AVOD participating hospitals (Table 3). The proportion of patients treated with antibiotics ranged from 4 to 63% across all hospitals (Fig. 1A), and rates of management with hospitalisation ranged from 12 to 46% (Fig. 1B). Among AVOD hospitals, the mean proportion of patients treated with antibiotics was 7%, compared to 38% among non-AVOD hospitals (*p* < 0.001). Accordingly, the mean proportion of patients managed with hospitalisation was 18% at AVOD hospitals and 38% at non-AVOD hospitals (*p* < 0.001). The median duration of the antibiotic treatment for patients managed outpatient was 10 (IQR: 7–10) days at both AVOD and non-AVOD hospitals, and 5 (IQR: 4–5) versus 7 (IQR: 4–10) days at AVOD versus non-AVOD hospitals for hospitalised patients (Table 3).

**Table 3:** Treatment of patients at index hospital visit and information on revisits within 90 days

	AVOD ﻿overall	Danderyd	Norrköping	Västerås	Non-AVOD overall	Malmö	Södertälje	Torsby
	282 (63%)	123 (27%)	68 (15%)	91 (20%)	167 (37%)	86 (19%)	54 (12%)	27 (6%)
Management at index AUD visit								
No antibiotics	262 (93%)	113 (92%)	64 (94%)	85 (93%)	103 (62%)	32 (37%)	52 (96%)	19 (70%)
Outpatient	223 (79%)	88 (72%)	57 (84%)	78 (86%)	85 (51%)	30 (35%)	39 (72%)	16 (59%)
Hospitalisation	39 (14%)	25 (20%)	7 (10%)	7 (8%)	18 (11%)	2 (2%)	13 (24%)	3 (11%)
Antibiotics	20 (7%)	10 (8%)	4 (6%)	6 (7%)	64 (38%)	54 (63%)	2 (4%)	8 (30%)
Outpatient	9 (3%)	4 (3%)	3 (4%)	2 (2%)	19 (11%)	16 (19%)	-	3 (11%)
Duration (days)*	10 (7–10)	9 (7–10)	7 (7–9)	12 (11–13)	10 (7–10)	10 (9–10)	-	7 (6–7)
Hospitalisation	11 (4%)	6 (5%)	1 (2%)	4 (4%)	45 (27%)	38 (44%)	2 (4%)	5 (19%)
Duration (days)*	5 (4–5)	5 (3–5)	10 (10–10)	5 (4–6)	7 (4–10)	8 (5–10)	6 (3–8)	7 (2–7)

*N* (%), *Median (IQR)

Basic characteristics, assessed comorbidities, and risk factors at the index AUD visit were not statistically different between patients treated with or without antibiotics, nor between patients with outpatient management or hospitalisation. The median CRP was 63 (IQR: 30–93) mg/L for patients with outpatient management, and 91 (IQR: 50–141) mg/L for hospitalised patients (*p* < 0.001). The median CRP was 63 (IQR: 32–94) mg/L compared to 93 (IQR: 41–144) mg/L for patients treated with and without antibiotics respectively (*p* < 0.001).

### 90 days follow-up

Within the 90-day follow-up period, 51 (11%) patients had a revisit of whom 5 patients, 1% of all, had developed complicated diverticulitis. The median day of the revisit was day 7 (IQR: 3–20), and all 5 patients with complicated diverticulitis revisited within 25 days (median day 19 (IQR: 9–21)). At the revisit, 5 (14%) patients at AVOD hospitals and 4 (31%) patients at non-AVOD hospitals were treated with antibiotics. Hospitalised patients at the revisit constituted 11 (30%) and 3 (22%) patients at AVOD and non-AVOD hospitals, respectively (Table 4). Of all revisits, 44 (86%) were within 30 days of the index AUD.

**Table 4:** Patients with revisit

	AVOD overall	Danderyd	Norrköping	Västerås	Non-AVOD overall	Malmö	Södertälje	Torsby
	282 (63%)	123 (27%)	68 (15%)	91 (20%)	167 (37%)	86 (19%)	54 (12%)	27 (6%)
Revisit within 90 days								
No	244 (87%)	108 (88%)	57 (84%)	79 (87%)	154 (92%)	84 (98%)	46 (85%)	24 (89%)
Yes	38 (13%)	15 (12%)	11 (16%)	12 (13%)	13 (8%)	2 (2%)	8 (15%)	3 (11%)
Diagnosis at revisit								
AUD confirmed	35 (13%)	13 (11%)	11 (16%)	11 (12%)	11 (7%)	2 (2%)	7 (13%)	2 (7%)
Complicated AD	3 (1%)	2 (2%)	-	1 (1%)	2 (1%)	-	1 (2%)	1 (4%)
Management at revisit								
No antibiotics	32 (86%)	11 (73%)	11 (100%)	11 (92%)	9 (69%)	2 (100%)	5 (63%)	2 (67%)
Outpatient	25 (68%)	9 (60%)	8 (80%)	8 (67%)	8 (62%)	2 (100%)	4 (50%)	2 (67%)
Hospitalisation	7 (19%)	2 (13%)	2 (20%)	3 (25%)	1 (7%)	-	1 (13%)	-
Antibiotics	5 (14%)	4 (27%)	-	1 (8%)	4 (31%)	-	3 (38%)	1 (33%)
Outpatient	1 (3%)	1 (7%)	-	-	2 (15%)	-	2 (25%)	-
Hospitalisation	4 (11%)	3 (20%)	-	1 (8%)	2 (15%)	-	1 (13%)	1 (33%)

*N* (%)

Of the 365 patients treated without antibiotics, 46 (13%) had a revisit within 90 days, compared to 6% of patients treated with antibiotics (*p* = 0.083). The median CRP at the index AUD visit was 67 (IQR: 39–108) mg/L compared to 66 (IQR: 32–103) mg/L for patients with and without revisit within 90 days, respectively (*p* = 0.83).

The rate of patients with revisit within 90 days did not differ significantly between AVOD hospitals (13%) and non-AVOD hospitals (8%) (Table 3). Notably, at one hospital, only 2% of patients had a revisit within 90 days, whereas 11–16% of patients from all other hospitals had a revisit within 90 days.

There were no deaths during the follow-up period, and no patients were diagnosed with colorectal cancer (CRC) during the 90-day follow-up. Overall, 230 patients (51%) were referred to follow-up colonic investigations (colonoscopy or CT-colonography).

### CT-verified AUD

The subgroup analysis included 369 (82%) patients with CT-verified AUD. In this subgroup, the proportion of patients treated with antibiotics was lower at AVOD versus non-AVOD hospitals (7% versus 43%, *p* < 0.001). Similarly, the proportion of patients managed with hospitalisation was lower at AVOD hospitals compared to non-AVOD hospitals (20% versus 46%, *p* < 0.001).

## Discussion

In this study, most patients with AUD were managed without antibiotics in an outpatient setting according to guidelines, although there were noteworthy variances depending on previous participation in the AVOD trial. AVOD hospitals showed greater adherence to the guidelines.

This study adds to the evidence of managing AUD outpatient without antibiotics and strengthens the importance of adhering to clinical guidelines resulting in cost-effective healthcare [15]. The findings underline the necessity of focusing on barriers towards change in clinical practice. Outpatient management of AUD without antibiotics is in general considered safe. Despite clear recommendations in current guidelines for AUD management, implementation remains lacking. This may be due to established routines and a mental barrier to changing practice. Interestingly, we found that hospitals previously involved in a clinical trial adhered more to guidelines than those that had not participated. This suggests a higher likelihood of adherence to evidence-based clinical management among professionals and healthcare systems involved in clinical research. One of the non-AVOD hospitals had a proportion of patients treated with antibiotics like the AVOD hospitals. This implies that updated guidelines had been adopted, potentially due to the interest in AUD among certain healthcare professionals positively influencing local practices.

A 2022 Cochrane review highlighted that, although increasing evidence supports treating AUD without antibiotics, the overall body of evidence remains limited [21]. The current study was not designed to challenge current guidelines but yield some noteworthy findings. The rate of emergency revisit within 90 days among patients treated without antibiotics was 13%, higher than the 3% reported in other studies of outpatient management without antibiotics [17], but comparable to the 16% within 1 year and 18% within 6 months reported in the RCTs comparing antibiotic versus no-antibiotic treatment [9, 10]. Notably, only 1% of patients in the current study developed complicated diverticulitis, a rate that was lower than in comparable studies [9, 10, 12]. This rate was not associated with treatment at the index AUD visit. One hospital with 86 patients had only 2% of patients with a revisit within 90 days. This hospital also had the highest proportion of patients treated with antibiotics (63%) and the second-highest proportion of hospitalised patients (46%). Although these numbers are intriguing, they may represent an outlier rather than a trend in the current study.

Hospitalisations for diverticulitis are an increasingly substantial financial burden on healthcare services [2, 22, 23]. Outpatient management without antibiotics is cost-effective. In the current study, the median length of stay in the 152 hospitalised patients was 5 days, adding up to a total of 760 days of hospitalisation. The 2014 Spanish DIVER trial found that treating AUD patients as outpatients, even with the administration of oral antibiotics, saved €1124.70 per patient compared to hospitalisation [15]. With healthcare spending universally on the rise, there is a continuous focus on providing cost-effective care, and the potential savings in managing AUD should not be overlooked.

In the current study, the median CRP was higher for hospitalised patients than for those managed as outpatients and higher for patients treated with antibiotics compared to those not treated with antibiotics. Previous studies have found CRP to be an important parameter for predicting the risk of complicated diverticulitis, although a predictive cutoff value (range 93–175 mg/L) has not been agreed upon [24, 25]. We found that CRP at index AUD visit did not differ between patients with or without revisit within 90 days, but our study was underpowered to estimate the predictive value of the CRP level in AUD.

Patients were included both before and after the COVID pandemic hit Sweden during the spring 2020. Resource reallocations were performed [26], which potentially encouraged outpatient management of patients suffering from AUD and may impact the use of antibiotics. We argue that it would affect the participating hospitals similarly and therefore not influence the findings.

The current study was based on retrospective data with minimal missing data. Selection bias was unlikely since all consecutive patients were included and there was no loss to follow-up. Limitations in the current study include the diagnostic accuracy of the patients included. According to current guidelines, CT is recommended to confirm the diagnosis in patients with no prior diagnostic information [7]. Although most patients in our study had CT-verified AUD, 7% of patients neither had a history of diverticulitis nor had a CT performed. AVOD hospitals had a higher proportion of patients undergoing CT, which may be attributed to greater adherence to guidelines overall, not just regarding the primary focus of the AVOD trial. Notably, we found the primary outcome of treatment of AUD compared between AVOD and non-AVOD hospitals to be consistent in the subanalysis of only patients with CT-verified AUD. Another limitation is potential unmeasured confounding variables. The external validity could have been improved with the participation of more hospitals, and the inclusion of a larger patient population. A longer follow-up period would allow for the investigation of long-term outcomes.

## Conclusions

Although most patients with AUD were managed according to guidelines in this study, there were large differences across hospitals. Hospitals that had previously participated in the AVOD study showed greater adherence to the guidelines regarding management of AUD. This suggests that previous participation in clinical studies may increase knowledge of and adherence to guidelines for managing AUD.

## Supplementary Information

Below is the link to the electronic supplementary material.

Supplementary file1 (DOCX 34.3 KB)

## Data Availability

No datasets were generated or analysed during the current study.
